# Teaching and Learning Medical Biochemistry: Perspectives from a Student and an Educator

**DOI:** 10.1007/s40670-014-0004-7

**Published:** 2014-05-07

**Authors:** Mehdi Afshar, Zhiyong Han

**Affiliations:** Department of Biochemistry and Molecular Medicine, the George Washington University School of Medicine and Health Sciences, Room 547, Ross Hall, 2300 Eye Street NW, Washington, DC 20037 USA

Advancement of medicine and that of biochemistry are inseparable, and much of modern medicine would not be practiced in the ways, as they are known today, without our understanding of how genetic, pathogenic and environmental factors affect the human body at the biochemical level. Thus, the importance of teaching medical students biochemistry is self-evident. Ironically, many medical students and practicing physicians consider learning biochemistry an unnecessary burden and that biochemistry has very little relevance to their daily practice of medicine [1–3]. Also, many students, especially those interested in fields such as primary care or psychiatry, also complain that there is too much anatomy in the preclinical curricula [1]. Thus, it seems that these students would prefer to selectively learn subject matters that they believe to be relevant to the medical specialties that they wish to acquire and practice in. Such utilitarian thinking, we believe, is in part responsible for the trend in medical curriculum that devaluates basic science and emphasizes apprenticeship experiences with clinical faculty [4].

Why do these medical students consider it unnecessary to learn biochemistry? What can educators do to convince them that learning biochemistry is important in their education? We have tried to answer these questions from the perspectives of M.A. (Mehdi Afshar), a fourth year medical student, and Z.H. (Zhiyong Han), a medical biochemistry educator. Based on our own experiences, we have considered and discussed four factors that we think partly explain why some medical students have unfavorable opinions about biochemistry (Table 1). We hope that our discussions could stimulate similar discussions among medical students and biochemistry educators elsewhere.

**Table 1:** Factors negatively affecting medical students’ interest in biochemistry

1. Medical biochemistry often repeats the materials of undergraduate biochemistry
2. Medical biochemistry is presented mostly outside the context of medical relevance
3. There is a large portion of the material that seems irrelevant to board examinations
4. Medical biochemistry requires too much rote memorization that does not last long

## Medical Biochemistry Needs to Present New Biochemistry Knowledge

A large number of the students admitted to medical schools already have undergraduate biochemistry education. But the experiences of MA show that much of his medical biochemistry materials are unnecessarily taught in ways and depth similar to those in his undergraduate biochemistry courses. We suggest that medical biochemistry should incorporate students’ previously learned knowledge with medical applications and fill in the gaps with new knowledge. For example, instead of re-teaching medical students to compare and contrast the structures of DNA and RNA and ask them to explain the difference between bases, one could discuss the biochemical basis of the “omics” that are profoundly changing medical research and medicine. Also, instead of re-teaching medical students the glycolysis pathway and asking them to memorize minute details of it, one could discuss the Warburg effect and why the glycolysis pathway serves to produce not only ATP, but more importantly molecules that are important for the metabolic requirements of cancer cells [5]. One could even discuss the rational for selecting certain glycolysis enzymes for the development of anti-cancer drugs [6]. This way of teaching is very likely to get students excited because it teaches them new knowledge and the applications of biochemistry knowledge to not only today’s medicine, but also future medicine.

## Medical Students Need to Learn Medically Relevant Biochemistry

It puzzles students that medical biochemistry is generally presented outside the context of diseases and medicine. For example, medical students are still taught how to use the Gibbs free energy equation to calculate the free energy and equilibrium constant of reactions. We fail to see medical relevance of being able or unable to do so. Therefore, like others [7], we also suggest that educators need to focus on teaching medical biochemistry in ways that show medical relevance. To us, medically relevant biochemistry is one that gives students just enough information to be able to understand the basic mechanism of why a biochemical defect results in a disease and potential avenues of diagnosis and treatment. We admit that this is not an easy task, because so much biochemistry is about chemical formula, reaction mechanisms, pathways, and seemingly unrelated schemes that are too far detached from living human body functions, such as blood flow or heart beats; furthermore, because medical students take biochemistry prior to having any medical knowledge and hence it is extremely difficult for them to relate biochemistry to diseases by themselves as MA can tell from his personal experience. Nevertheless, it appears that most students can effectively learn and understand medical applications of biochemistry if the applications are presented to them in contextualized ways through uncomplicated medical cases. For example, students could easily see the biological importance and medical relevance of the *V*_max_ and *k*_m_ of glucokinase if they are shown real life cases of diabetes caused by mutations that alter the *V*_max_ and *k*_m_ of glucokinase [8].

Therefore, we suggest a way of teaching medical biochemistry. To do so, medical biochemistry educators need first to define core concepts and biochemical principles that teachers and students could review (or relearn) in a short period. The educator will then teach new concepts and medical applications of biochemistry, in an in-depth fashion, via a series of carefully designed PBL (problem-based learning) or CBL (case-based learning) modules [9]. The in-depth teaching should aim to (1) stimulate personal cognitive processes to first understand concepts and then connect concepts to construct knowledge structures, and (2) teach students to apply their knowledge to new situations and solve new problems. This way of teaching promotes lifelong learning, open inquiry, and critical thinking capability that physicians should have [10, 11].

Alternatively, medical biochemistry could be taught in various way that integrate basic and clinical sciences that have been or are being adapted by medical schools worldwide [12–14]. Imagine the following scenario in an integrative curriculum. A biochemist starts the day by teaching students the biological roles of cholesterol, its synthesis, absorption, transportation, and disposition and how alterations in any of these processes change the laboratory values of the blood lipid test. The pathologist then continues by teaching the disease processes that result from dysregulation of the aforementioned events involving cholesterol. The nutritionist follows up by teaching dietary management of cholesterol-related disorders. Finally, the pharmacologist ends by discussing various treatment options and potential for future therapies. This integrative teaching allows students to see cholesterol metabolism in relationship to diseases and medicine from different perspectives without any delay.

## Teaching of Medical Biochemistry Should Not Revolve Around Board Relevance

Some students seem to like the idea of learning just “board examination relevant” materials. As such, they simply want biochemistry to be presented to them in bullet points format that shows “key words connections” that are easy to recall during board examinations. This is a bad idea. Since board examinations assess a physician’s minimum competencies, we should not set the bar to the height of minimum competencies and limit ourselves to teaching and learning just a minimal amount of materials for passing examinations. We believe, for example, that it is not enough that physicians prescribe nucleoside analog-derived drugs, such as AZT, to treat HIV/AIDs—they should be able to articulate the biochemistry underlining the action mechanisms of these drugs. What we do *not* want is to produce physicians who can pass the board examinations with knowledge deficiencies and who are unable to critically read and understand the sciences in the articles published in medical journals, such as the *Journal of Clinical Investigation*, *The Lancet*, or the *New England Journal of Medicine*.

## We Must Minimize Rote Memorization of Materials and Make Information Stick

Needless to say, students can attest that they have to memorize a huge amount of biochemistry materials. However, rote memorization is not synonymous to learning and understanding of the materials and produces learning fatigue. In the era of using pocket devices to instantly gain access to ubiquitous information, we should stop asking students to memorize a vast amount of details, such as the minute details in the metabolic pathways, as long as it does not reduce the quality of educational outcome. Instead, we should teach students how to conceptualize metabolic pathways with an emphasis on the biological roles of metabolic pathways and their interconnection in the context of physiology and diseases. Then, we test the students’ understanding of the metabolic pathways and their interconnection by assessing whether they know where and what information to look for in the metabolic pathway charts when solving problems. Thus, it is best, for example, if a student knows how to diagnose a metabolic defect that causes methymalonic acidemia using a metabolic pathway reference. This idea was proposed by Professor Edward J. Wood 23 years ago, and he suggested then that it would be better if we stop “asking students to remember detailed information [of a metabolic pathway], to reproduce it accurately under stressful, time-limited [examinations] conditions, and in competition with their peers [15].”

Equally important, biochemistry needs to be taught in ways that stick with students—the stickier the better—to reduce rote memorization. There are many ways to make things stick [16], and it requires personalities, presentation styles, use of vivid and sticky examples, clarity, and even something that shocks [16]. For example, the shocking case of a 66-year-old “man” who turns out to be genetically a woman with Turner’s syndrome plus virilising 21-hydroxylase deficiency should allow students to visually understand the function and biological significance of the 21-hydroxlyase in the context of steroid synthesis [17]. Stories like this one should grab students’ attention, induce students’ curiosity and interest, unfold medical/biochemical investigative events before students, vividly relate biochemistry to medicine, and will stick!

In summary, medical biochemistry needs to highlight how biochemistry applies to medicine, minimizes rote memorization, and stick with students. The best outcome should be one that highlights the connection between medical biochemistry and its clinical applications. The ultimate goal of medical biochemistry, in our opinion, should be to provide students with fundamental biochemistry concepts and principles that serve as a knowledge foundation enabling them to better study and understand the complexities of diseases and medicine. Teaching biochemistry concepts and principles to students should be aimed at helping the students to become scientifically literate so that they will gain the ability to become independent learners in the future, and be able to critically read and understand biomedical literatures, participate in biomedical research projects, and evaluate claims of efficacy and safety of new therapeutic strategies. This will help students become the best doctors that they can be, and provides the best care for patients throughout their careers.
